# Systematic identification of lncRNA-based prognostic biomarkers for glioblastoma

**DOI:** 10.18632/aging.102393

**Published:** 2019-11-06

**Authors:** Mingdong Li, Shengrong Long, Jinqu Hu, Zan Wang, Chao Geng, Shaowu Ou

**Affiliations:** 1 Department of Neurosurgery, First Affiliated Hospital of China Medical University, Shenyang, China

**Keywords:** lncRNAs, copy number alterations, prognostic biomarkers, functional modules, glioblastoma

## Abstract

Glioblastoma (GBM), a primary malignant tumor of the central nervous system, has a very poor prognosis. Analysis of global GBM samples has revealed a variety of long non-coding RNAs (lncRNAs) associated with prognosis; nevertheless, there remains a lack of accurate prognostic markers. Using RNA-Seq, methylation, copy number variation (CNV), mutation and clinical follow-up data for GBM patients from The Cancer Genome Atlas, we performed univariate analysis, multi-cluster analysis, differential analysis of different subtypes of lncRNA and coding genes, weighted gene co-expression network analyses, gene set enrichment analysis, Kyoto Encyclopedia of Genes and Genomes analysis, Gene Ontology analysis, and lncRNA CNV analyses. Our analyses yielded five lncRNAs closely related to survival and prognosis for GBM. To verify the predictive role of these five lncRNAs on the prognosis of GBM patients, the corresponding RNA-seq data from Chinese Glioma Genome Atlas were downloaded and analyzed, and comparable results were obtained. The role of one lncRNA LINC00152 has been observed previously; the others are novel findings. Expression of these lncRNAs could become effective predictors of survival and potential prognostic biomarkers for patients with GBM.

## INTRODUCTION

GBM is one of the most common primary malignant tumors of the central nervous system, accounting for 47.1% of all brain malignant tumors. Information from the Central Brain Tumor Registry of the United States (CBTRUS) revealed that the incidence of malignant brain tumors increases with age (the highest incidence is in the over 85 years old age group [85.39 per 100,000 people]), while the lowest incidence is in children and adolescents aged 0–19 years old [5.76 per 100,000]. The incidence of GBM in the United States is about (3.20 per 100,000) [1] and the age of onset is mostly in the range of 45–70 years old. The cause of the disease remains unclear [2]. There are two scenarios of GBM onset: in the first scenario, existing low-grade gliomas evolve over time; in the second, more common scenario, GBM is identified at initial diagnosis [3–7]. GBM can be subdivided into four subtypes: classical, mesenchymal, neuronal and proneuronal, based on their transcriptional profiles [8, 9]. Standardized treatment options for GBM include surgical excision within the maximum safe range, post-operative adjuvant radiotherapy, and chemotherapy, most commonly with temozolomide, a cytotoxic alkylator.

Almost all GBMs eventually recur [10], and despite substantial advances in recent years, GBM still has a high mortality rate. The one-year survival rate after diagnosis is about 35.7% [11] and the survival time of more than three years is about 3%–5% [12]. The average median survival time is less than 15 months [13, 14]. The survival rate of GBM is inversely proportional to age. Specifically, about 5% of all GBM patients survive five years after diagnosis, while among the population over 65 years old, this proportion drops to 2% [15, 16]. The median survival time of untreated GBM patients is only about 3 months [17].

A better understanding of the genetic and molecular pathogenesis of GBM could yield more effective therapies. LncRNAs are long transcripts of 200 NT-100 kb that lack open reading frames (ORFs). They are involved in transcription, as well as epigenetic and post-transcriptional regulation, and play a role in tumorigenesis, invasion, metastasis and drug resistance of tumors [18–25]. They are usually transcribed by RNA polymerase II and controlled by transcriptional activators of SWI/SNF complexes. Most generated lncRNA transcripts are spliced, capped and polyadenylated in a manner similar to that of mRNAs [26–29]. The human genome contains more than 50,000 lncRNA genes [30], 584 of which have been associated with poor prognosis in a global analysis of GBM samples; 282 lncRNAs were associated with better survival in GBM patients and were confirmed to be prognostic biomarkers of GBM [31]. We sought to identify lncRNAs associated with the prognosis of GBM and to provide potential therapeutic targets for treatments by integrating RNA-Seq data, methylation data, CNV data, mutation data and clinical follow-up information.

## RESULTS

### Identification of mutation subtypes correlated to survival

**Figure 1:**
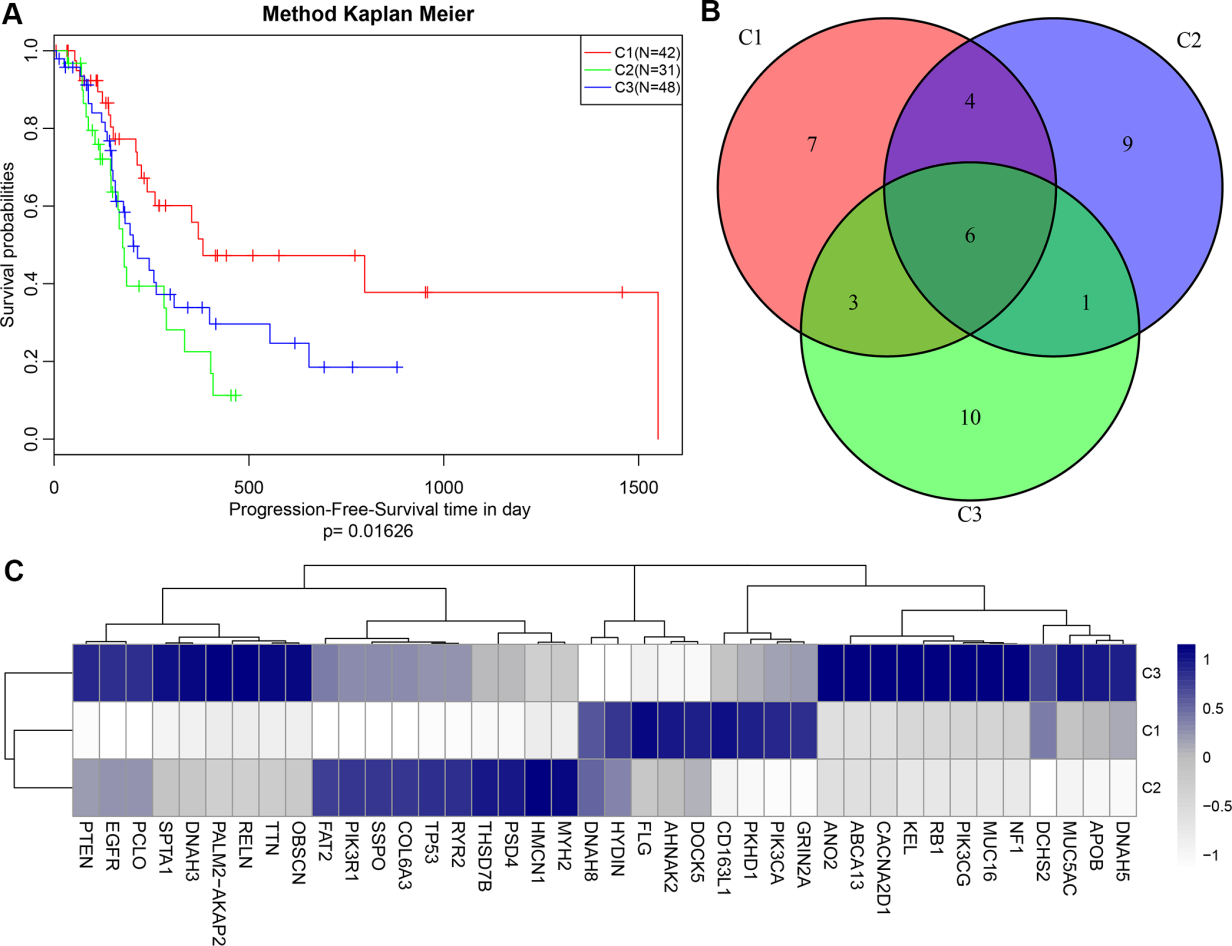
(**A**) Kaplan-Meier (KM) curves of disease-free survival (DFS) for three subtypes, p = 0.01626; (**B**) Venn plot of the top 40 genes with most frequent mutations in each subtype. 6 genes overlapped in all three subtypes; (**C**) Heat map of top 40 genes with the highest mutation frequency in each subtype.

A total of 1808 protein-coding genes, 8054 CNV regions and 4964 CpG loci were obtained using univariate Cox regression (see Methods). We set the number of categories for multi-omics cluster analysis to 3 and obtained three subtypes (Table 1). The sample classification for each subtype was relatively uniform. Analysis of these three subtypes (Figure 1) revealed significant prognostic differences among them (*p* = 0.01626). The C2 group had the worst prognosis, and the C1 group had the best. The top 20 genes with high mutation rates in each subtype were selected, and a total of 40 genes were obtained. The intersection of the top 20 genes with high mutation frequency in the three subtypes is shown in Figure 1B. The overlap of these genes among the three subtypes was minor. Mutation of these 40 genes in each subtype are visualized in Figure 1C, showing that their mutation frequencies were significantly different in each subtype, and the mutation frequencies of the samples in each subtype were also significantly different.

**Table 1:** Sample count in three different GBM subtypes.

**Cluster**	**Sample count**
C1	42
C2	32
C3	49

### Differential analysis of lncRNA and coding genes among different subtypes

**Figure 2:**
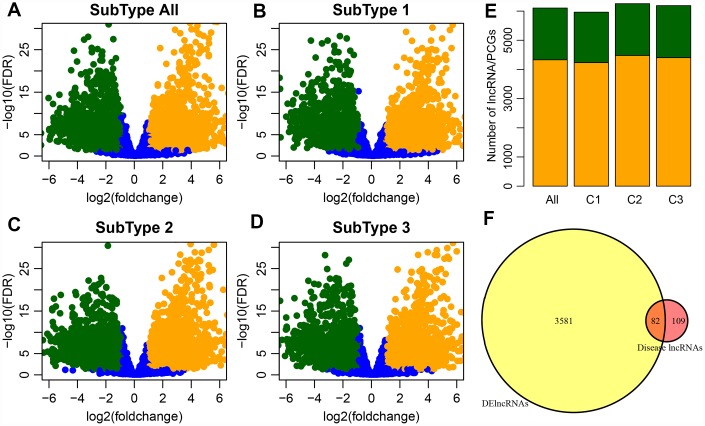
(**A**) Volcano plot shows the upregulated and downregulated lncRNAs in the GBM patients. The horizontal axis represents fold-change, whereas, the vertical axis represents the P value estimated by edgeR in GBM patient samples. (**B**–**D**) Volcano plots for DE-lncRNAs in the three GBM subtypes which correspond to three clusters (C1-C3) of GBM patients from integrative clustering of multiple data types. (**E**) Distribution of differential expression lncRNA and differential expression coding genes among groups, orange for genes, green for lncRNA. (**F**) Venn plot of disease-related lncRNA and differential lncRNA, p < 0.01.

**Figure 3:**
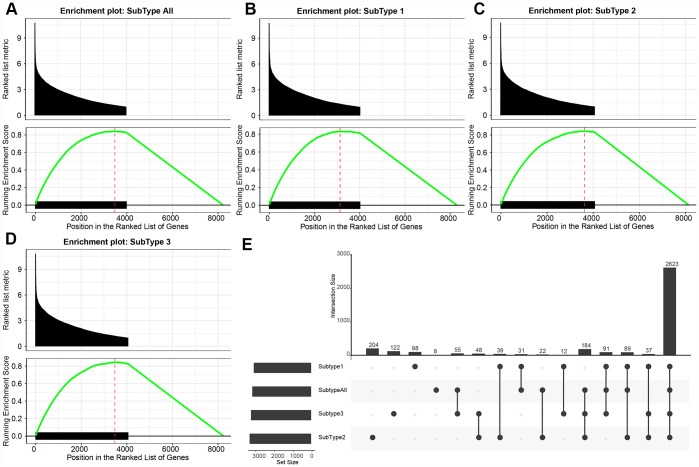
(**A**–**D**) GSEA plots shows the enrichment of all GBM and each subtype according to the fold-change as ranked. It can be seen that the DE-lncRNAs are enriched in the gene set with large differential fold. (**E**) The upset plot shows the intersection of three subtypes of DE- lncRNAs. There is a large overlap between the three subtypes and all tumor samples.

The numbers of differentially expressed genes and lncRNAs in each subtype were similar (Table 2). C2 samples possessed the most differential lncRNAs and genes, with a total of 3663 differential lncRNAs and 5057 coding genes. The volcano plot of differential lncRNAs among the subtypes is shown in Figure 2A–2D, suggesting that the number of the upregulated lncRNAs is generally greater than that of the downregulated lncRNAs. The numbers of differentially expressed lncRNAs and coding genes in each subtype were generally less than that of the coding genes (Figure 2E). Then, 611 lncRNAs closely related to the disease were downloaded from the database of LncRNA Disease and Lnc2Cancer and were compared with our 3663 lncRNAs with subtype differences (Figure 2F). Of these, 82 lncRNAs that were closely related to GBM were obtained. Significance was tested using the hypergeometric test (*p* < 0.001). Then, the lncRNAs were ranked based on the fold-change of their differential expression among various subtypes, and GSEA analysis was then performed (Figure 3A–3D). Differential lncRNA aggregated in gene sets with large multiples of difference. We analyzed intersections of lncRNAs, which have differential expression in GBM and normal samples and found a significant overlap of differential lncRNAs among the three subtypes and tumor samples (Figure 3E).

**Table 2:** Differentially expressed lncRNAs in GBM patients.

**Type**	**C1**	**C2**	**C3**	**All**
PCG_Down	1861	2056	1927	1867
PCG_Up	2375	2425	2479	2464
PCG_All	4236	4481	4406	4331
Lnc_Down	1293	1473	1390	1329
Lnc_Up	1727	1773	1782	1774
Lnc_All	3020	3246	3172	3103

The table shows the differentially expressed lncRNAs (DE-LncRNAs) and PCGs in the GBM patient group and the three GBM subtypes. Up and down arrows indicate upregulation and downregulation of differentially expressed genes, respectively. C1-C3 represent 3 clusters (clusters 1-3) of GBM patients based on integrative clustering of multiple lncRNA-related data in the underlying GBM subtypes.

### WGCNA analysis of coding genes and lncRNA with subtype differences

**Figure 4:**
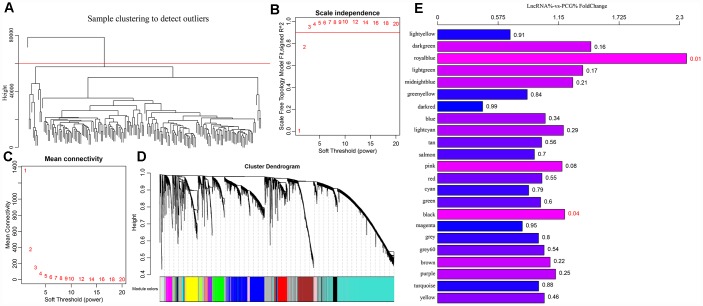
(**A**) GBM patients were classified into three clusters (C1-C3) which represent the underlying GBM subtypes by integrating multi-omics data using the iCluster. Differentially expressed lncRNAs and mRNAs in the GBM patients and the subtypes were identified. (**B**–**C**) Analysis of network topology for various soft-thresholding powers. (**D**) WGCNA analysis was then performed to identify co-expression lncRNA modules (M1−M23) using GBM transcriptome which contain GBM-related lncRNAs and mRNAs followed by functional enrichment analysis of the different modules. (**E**) The relative multiple histogram of lncRNA ratio and PCG ratio in 23 modules, the p value is on the right, the horizontal axis represents the multiple of lncRNA ratio and PCG ratio in the module, and the vertical axis represents the modules.

A cluster analysis of the samples (Figure 4A) eliminated outliers with distances greater than 60,000 and 171 samples were screened out. Then, Pearson correlation coefficients were used to calculate the distance between each gene and lncRNA. WGCNA analysis revealed a co-expression network that conformed to the scale-free network. That is, the logarithm of a node with connectivity K (log (k)) was negatively correlated with the logarithm of the occurrence probability of that node (log (P (k)), and the correlation coefficient was greater than 0.8. To ensure that the network was scale-free, ß was set to 3 (Figure 4B, 4C). Next, hierarchical clustering (average-linkage method) was used to cluster genes, and a total of 23 modules were obtained (Figure 4D). It should be kept in mind that the grey module could not be aggregated into the gene sets of other modules. Statistics of gene/lncRNA in each module are shown in Table 3 and Figure 4E, where the *p* value represents the significance of aggregation of a lncRNA in a module, and fc indicates the aggregation multiple. Significant enrichment of lncRNAs in black and royal blue modules were demonstrated.

**Table 3:** The genes and lncRNAs of each module.

**Module**	**All**	**Lnc**	**PCG**	**p.value**	**FC**
yellow	458	194	264	0.456169824	1.014504174
turquoise	2403	986	1417	0.877387079	0.960645383
purple	174	78	96	0.246399739	1.121706934
brown	568	248	320	0.216710599	1.069935845
grey60	52	22	30	0.535556439	1.012412412
grey	1356	556	800	0.80085116	0.959490854
magenta	228	84	144	0.953173448	0.805328055
black	360	168	192	0.038356998	1.207992083
green	424	176	248	0.602914984	0.979753947
cyan	96	37	59	0.7862313	0.865776408
red	385	161	224	0.550523559	0.992279211
pink	299	138	161	0.078357402	1.183339183
salmon	110	44	66	0.699306048	0.92037492
tan	115	48	67	0.559107858	0.989059616
lightcyan	56	26	30	0.294146357	1.196487396
blue	1245	530	715	0.342746608	1.023353932
darkred	38	9	29	0.994428137	0.428450394
greenyellow	131	50	81	0.837942895	0.852199
midnightblue	56	27	29	0.208831656	1.285351182
lightgreen	46	23	23	0.170505202	1.380562381
royalblue	38	24	14	0.006833389	2.366678367
darkgreen	37	19	18	0.161718128	1.457260291
lightyellow	45	15	30	0.910168528	0.69028119

**Figure 5:**
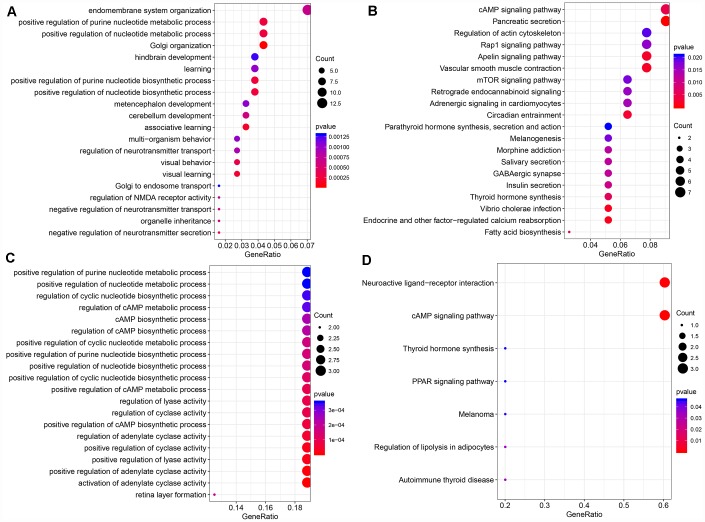
(**A**) The bubble plot shows the results of GO enrichment of top 20 of Black Module. (**B**) Results of KEGG enrichment of top20 of Black Module. (**C**) Results of GO enrichment of top20 of Royal blue Module. (**D**) Results of KEGG enrichment of top7 of Royal blue Module**.**

The function of modules with significant lncRNA enrichment was then analyzed. 247 Pathway and GO terms were enriched from the two modules; a minor intersection of 10 (4%) was found when analyzing their cross talk. They tended to be enriched in different pathways, suggesting that different functions might be performed by different modules. The first 20 GO Terms enriched by black module (Figure 5A) were related to transcriptional activation. These 20 enriched pathways were related to metabolic and mTOR signaling pathways (Figure 5B). The first 20 GO terms enriched by royal blue pathway were associated with cAMP signal transduction (Figure 5C). Seven KEGG pathways were enriched by royal blue pathway and the most significant pathways were the cAMP signaling pathway and neuroactive ligand-receptor interaction (Figure 5D).

### CNV analysis of lncRNA

**Figure 6:**
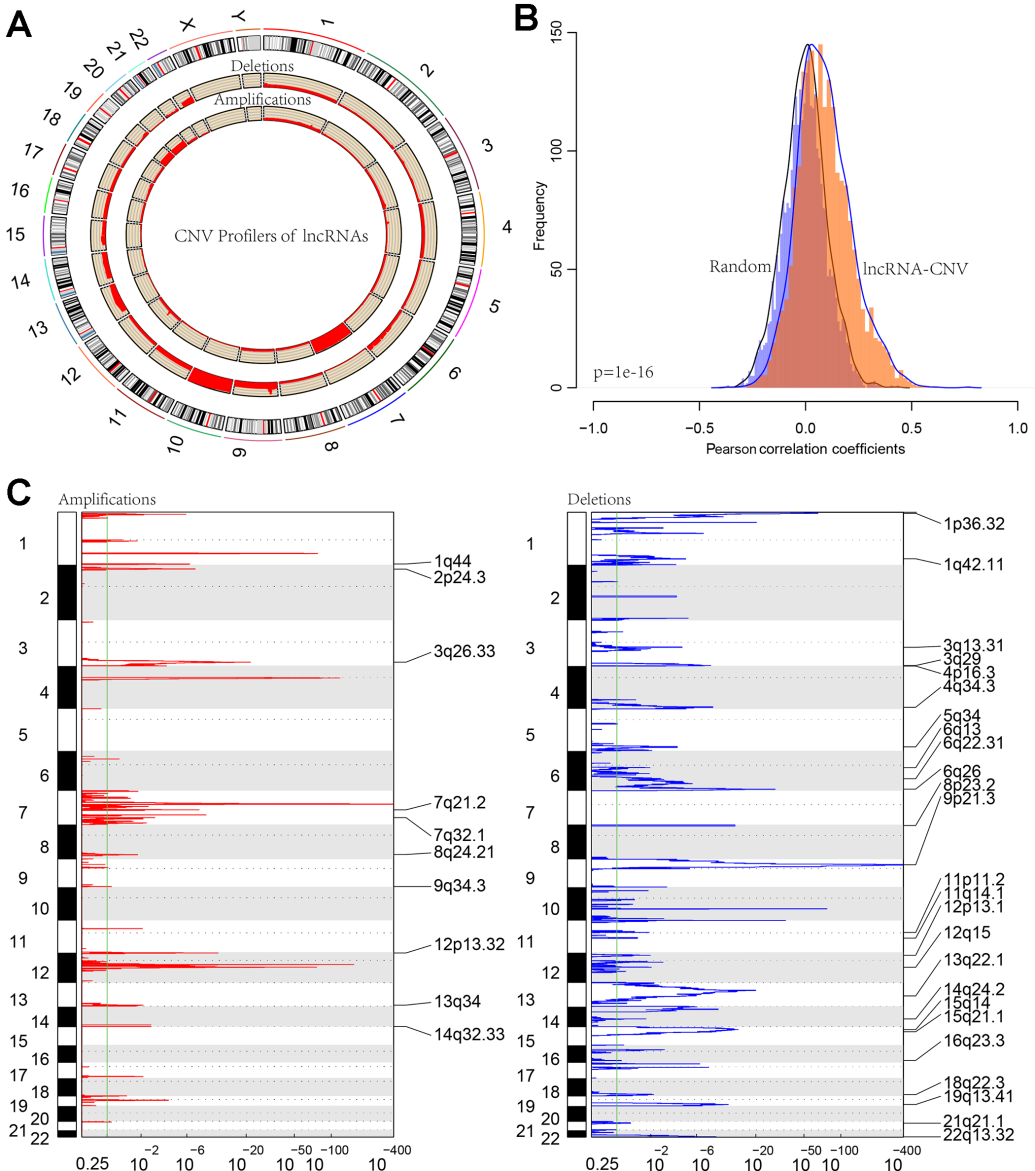
(**A**) CIRCOS plot shows the genome-wide view of CNV in lncRNA genes. The outer ring sections represent the chromosomes. Each section/chromosome size is relative to each other. The CIRCOS plot is divided into three tracks. The histogram in the outer track displays the CNV of the GBM-related lncRNAs. The two inner tracks display amplification and deletion of chromosome. (**B**) The distribution of Pearson correlation coefficients between copy number and expression profiles of lncRNAs. (**C**) The lncRNAs located in the focal CNA peaks are GBM-related. FDRs (q values) and scores from GISTIC 2.0 for alterations (x-axis) are plotted against genome positions (y-axis); dotted lines indicate the centromeres. The amplifications and deletions of lncRNAs are also shown.

Distribution of copy deletion and copy amplification of lncRNAs in genome is shown in Figure 6A. Copy amplification was significantly less frequent than copy deletion. The proportion of deletion was the highest in chromosome 10 while the proportion of amplification was the highest in chromosome 7.

Furthermore, correlation distribution between the expression profiles of the lncRNAs and copy numbers was calculated (Figure 6B). An overall positive correlation trend between copy number and lncRNA expression was indicated, the distribution of which was significantly higher than random (*p* < 1e-16). Frequently changing regions in the GBM genome were identified using the GISTIC algorithm, and multiple regions with significant multicopies or copy deletions of lncRNAs were identified. Frequent copy deletions of lncRNAs were significantly more abundant than those of the amplified regions (Figure 6C), suggesting that the deletion of lncRNA copy may be related to the occurrence and development of GBM.

**Figure 7:**
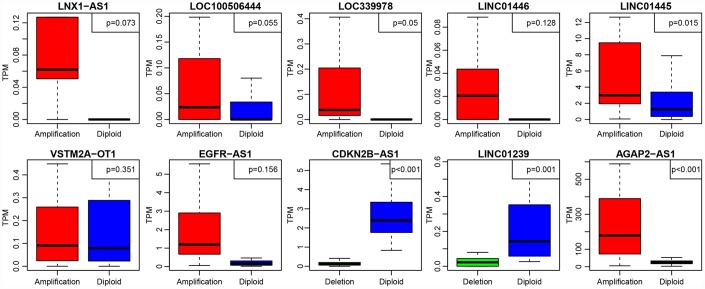
**The expression of 10 lncRNAs with deletions and amplifications of genes in GBM patients.** The P values show the significant level of the correlation coefficients between copy number variation and lncRNA expression.

To further investigate the relationship between expression levels of lncRNAs and copy numbers, fifteen lncRNAs with a copy ratio of more than 10% in each sample were selected. Expression differences of each lncRNA in the samples with amplified, deleted and normal copy numbers were analyzed. No fewer than ten samples with expression levels greater than zero in each group were selected and thus ten lncRNAs were obtained (Figure 7). Eight (80%) showed significantly higher expression in the copy-amplified samples than in the normal ones. Two reached significant differences, and another three had marginally significant differences. Two lncRNAs (20%) showed significantly lower expression in the copy-deleted samples than in the normal ones, suggesting that CNV was closely related to the expression of lncRNAs.

### LncRNA-based prognostic biomarkers in GBM patients

We found a total of 172 differentially expressed lncRNAs with co-expression module of the gene expression. A total of 13 (59%) lncRNAs showed differences of expression among the three subtypes (Table 4).

**Table 4:** 22 GBM-related, differentially-expressed and potentially prognostic lncRNAs.

**LncRNA**	**p.value**	**HR**	**Low 95%CI**	**High 95%CI**	**CNV.Rate**	**DECount**	**Module**	**Symbol**
ENSG00000186056	0.030317	1.49	1.038648	2.137489	0.005034	4	brown	MATN1-AS1
ENSG00000236268	0.044121	1.582918	1.01216	2.475527	0.001678	4	turquoise	LINC01361
ENSG00000228203	0.019549	1.15539	1.023464	1.304322	0.005034	4	turquoise	RNF144A-AS1
ENSG00000222041	0.015702	1.011345	1.002131	1.020644	0.001678	4	black	LINC00152
ENSG00000225539	0.047346	5.01846	1.019074	24.71356	0.003356	4	turquoise	LOC101927406
ENSG00000240875	0.035074	1.095223	1.006388	1.1919	0.003356	2	magenta	LINC00886
ENSG00000234111	0.043678	239.1842	1.168031	48979.07	0.003356	4	turquoise	LOC340017
ENSG00000248859	0.000137	1.395472	1.175857	1.656105	0.005034	4	turquoise	LINC01574
ENSG00000224596	3.19E-05	1.505299	1.241456	1.825217	0.006711	2	yellow	ZMIZ1-AS1
ENSG00000221949	0.009864	1.215273	1.048005	1.409238	0.011745	1	lightcyan	LINC01465
ENSG00000251301	0.017577	1.328054	1.050768	1.678512	0.025168	3	tan	LOC100507195
ENSG00000246363	0.018556	1.784352	1.101855	2.88959	0.003356	4	turquoise	LOC728084
ENSG00000238121	0.04526	2.387149	1.018536	5.594775	0.006711	3	yellow	LINC00426
ENSG00000259062	0.045396	1.415307	1.007143	1.988889	0.001678	4	lightcyan	ACTN1-AS1
ENSG00000261801	0.00024	1.120072	1.054312	1.189934	0.001678	1	cyan	LOXL1-AS1
ENSG00000259234	0.036685	3.588288	1.082273	11.89701	0.001678	4	turquoise	ANKRD34C-AS1
ENSG00000277639	0.016627	1.046485	1.008285	1.086132	0.001678	4	magenta	LOC105371267
ENSG00000179219	0.026907	0.491178	0.261676	0.921963	0.005034	4	purple	LINC00311
ENSG00000267532	0.020133	1.030549	1.004723	1.057038	0.001678	2	lightcyan	MIR497HG
ENSG00000263400	0.030058	1.064975	1.006094	1.127303	0.001678	1	magenta	TMEM220-AS1
ENSG00000264235	0.012691	1.219032	1.043212	1.424484	0.003356	2	magenta	LOC104968399
ENSG00000231290	0.00454	1.123154	1.036584	1.216955	0.005034	4	cyan	APCDD1L-AS1

**Figure 8:**
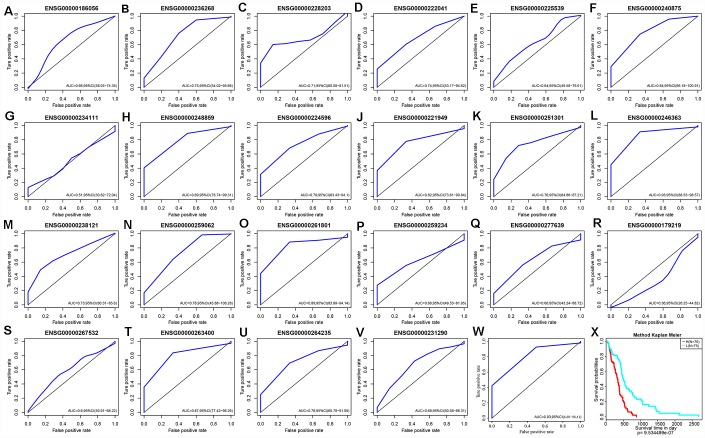
(**A**–**V**) ROC curves of 22 lncRNAs with significant prognostic value for GBM. (**W**) ROC curves of five-lncRNA model. (**X**) Patients of High-Risk and Low-Risk group. Survival analysis showed that the prognosis of the high-risk group was worse than that of the low-risk group, p < 0.01.

Based on expression levels of these 22 lncRNAs in each sample, their efficacies in prognostic classification were analyzed; the corresponding ROC curves are shown in Figure 8A–8V. Most had higher AUCs in their prognostic classifications, with an average of 0.727. Thirteen lncRNAs with AUCs greater than the average were selected. Multivariate survival analysis revealed substantial interaction among the 13. Using stepwise multivariate regression, five lncRNAs were screened out as independent prognostic factors (Table 5). A risk score for each sample was calculated according to the multivariate regression model composed of the five lncRNAs (Risk Score=0.01*ENSG00000222041+0.36* ENSG00000248859+0.3*ENSG00000224596+0.09*ENSG00000261801+0.07*ENSG00000263400). Then, all samples were divided into high-risk and low-risk groups based on the score. The ROC curves of the risk models for the five lncRNAs had AUC of 0.93 (Figure 8W). All cases were divided into the high- and low-expression groups according to the median of the risk score. Survival analysis with Kaplan-Meier curves suggested that the prognosis of the high-risk group was significantly worse than that of the low-risk group (Figure 8X).

**Table 5:** Five independent prognostic CNV-related lncRNAs.

**lncRNA**	**Symbol**	**exp(coef)**	**lower .95**	**upper .95**	**z**	**Pr(>|z|)**
ENSG00000222041	LINC00152	1.009198434	0.998988854	1.019512355	1.764959658	0.077570546
ENSG00000248859	LINC01574	1.438491798	1.200262175	1.724005551	3.93600151	8.29E-05
ENSG00000224596	ZMIZ1-AS1	1.356509194	1.056254919	1.742114673	2.388718419	0.016907255
ENSG00000261801	LOXL1-AS1	1.095661366	1.020034879	1.176894882	2.503570315	0.012294725
ENSG00000263400	TMEM220-AS1	1.067615892	1.001915016	1.137625123	2.019001738	0.043487039

**Figure 9:**
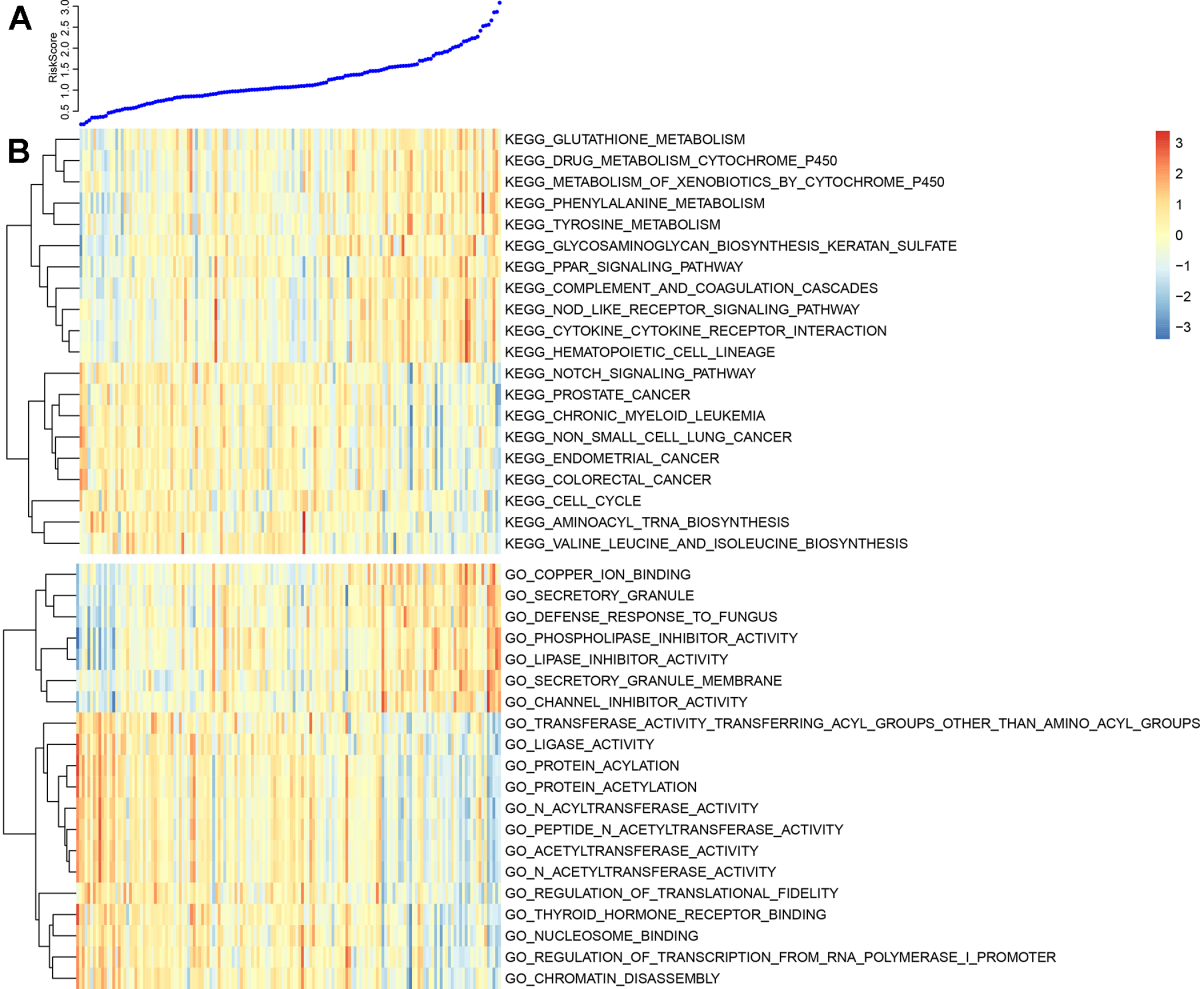
(**A**) The top curve shows Risk Score distribution of GBM patients. (**B**) The heat map of Risk Score-related top 20 KEGG Pathway and top 20 GO Terms.

Enrichment analysis was performed using the KEGG pathways and GO terms of each sample using gene expression profiles and ssGSEA. The top 20 KEGG pathways and GO terms correlating with risk scores were screened out. The average correlation coefficient of the top 20 KEGG pathways was 0.37 (Figure 9A). Nine had negative correlations, several of which were associated with cancer. Another 11 pathways positively correlated with metabolic processes. The average correlation coefficient of the top 20 GO terms was 0.5 (Figure 9B). Of these, 13 were negatively correlated and 7 were positively correlated. Conclusively, the prognostic risk score predicted by these five lncRNAs was closely related to the development of cancer.

### Validation of external data sets

**Figure 10:**
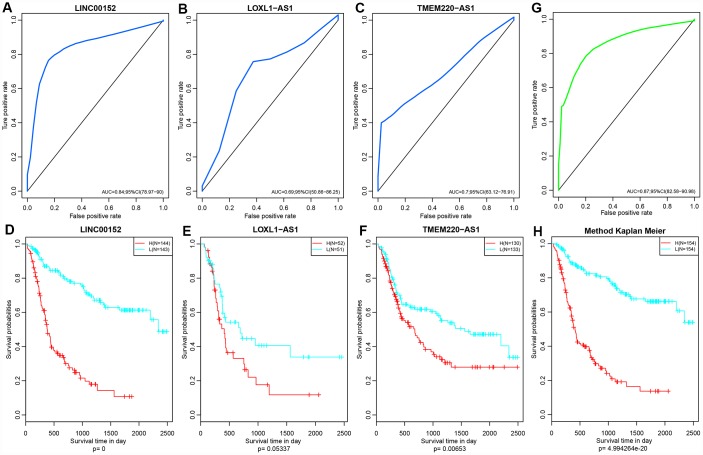
(**A**–**C**) ROC curves of three lncRNAs: LINC00152, LOXL1-AS1 and TMEM220-AS1. (**D**–**F**) KM Curves of these three lncRNAs. (**G**) ROC curves of this three-lncRNA model/panel. (**H**) The prognostic difference KM curve of the samples predicted by three lncRNA-models. Prognostic differences among them suggested that the prognosis of the high-risk group was significantly worse than that of the low-risk group (*p* < 0.0001

Five lncRNAs were compared with the CGGA dataset, and we determined expression profiles for three of these. Prognosis was predicted based on expression levels of these three lncRNAs. Only three of five lncRNAs that are detected in CGGA database due to technical limitations. The ROC curve showed that all three lncRNAs had high AUCs, similar to that of the training set (Figure 10A–10C). All cases were divided into groups according to the median of the expression level. Differences of prognosis between high- and low-expression groups were analyzed. As shown in Figure 10D–10F, there were significant prognostic differences between these two group (*p* <0.05) in two lncRNAs and the other one showed a marginally significant difference (*p* = 0.05337), consistent with the training set. Based on the expression profiles of these three lncRNAs, a risk prediction model was established using multivariate Cox regression and a prognostic risk score of each sample was calculated. The AUC of the ROC curve was 0.87 (Figure 10G). Then, all patients were divided into high- or low-risk groups based on the median of the risk score. Prognostic differences among them suggested that the prognosis of the high-risk group was significantly worse than that of the low-risk group (*p* < 0.0001) (Figure 10H).

### Comparison with other known prognostic biomarkers

**Figure 11:**
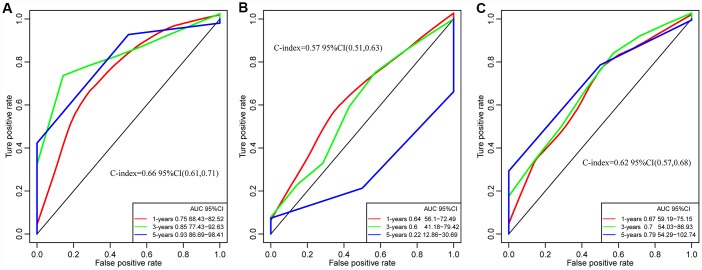
**The ROC curves of of 1 year, 3 years and 5 years in studies.** (**A**) Our study. C-index = 0.66. (**B**) Cheng's study. C-index= 0.57. (**C**) Guo's Study. C-index = 0.62. Our Signatures is better than the other two signatures in ROC and C-index.

In order to evaluate whether our prediction model has a stable and reliable performance advantage, we compared two recent studies on gene markers related to the survival and prognosis of GBM. The ROC curves of 1 year, 3 years and 5 years data were analyzed. The AUC of our model in 3 years was 0.85, the highest was 0.93, the smallest AUC was 0.75 such as Figure 11A, and the highest AUC of 8 immune gene signature (Cheng et al) was 0.64 such as Figure 11B [32]. The highest AUC of 4 gene panel of Guo et al was 0.79 (Figure 11C) [33]. On the other hand, comparing the C-index of the three models, our model has the highest C-index, 0.66, and our model is better than the other two models in all aspects. All of these results provide an exciting revelation that our study provides a better predictive model for predicting overall survival (OS) in patients with GBM.

## DISCUSSION

LncRNAs have become new biomarkers for the diagnosis and prognosis of various human cancers [34–37]. We speculated that differential expression and CNV of lncRNAs may serve as prognostic biomarkers for patients with GBM. Therefore, we identified lncRNAs indicating potential risk of GBM patients using a multi-omics approach and by integrating the expression and CNV of lncRNAs. Several lncRNAs, including MEG3, H19 and Gas5, were abnormally expressed in all GBM patients [38–41]. Some GBM-specific lncRNAs were expressed differentially in various GBM subtypes. A lncRNA-PCG co-expression module was identified in GBM patients. These differentially expressed lncRNAs are involved in the regulation of critical biological functions associated with cancer. For example, ENSG00000231327 (LINC01816) in the black module is related to transcriptional activation, metabolism and mTOR signaling pathways. In addition, two differentially expressed lncRNAs in the royal blue module ENSG00000266088 (Rp5-1028K7.2) and ENSG00000234184 (LINC01781) were associated with cAMP signaling and interactions with the neuroactive ligand-receptor.

We identified five lncRNA biomarkers associated with prognosis of GBM patients, LINC00152, LINC01574, ZMIZ1-AS1, LOXL1-AS1 and TMEM220-AS1, all of which were highly expressed in GBM and correlated with lower OS (*p* = 0). The association between LINC00152 and GBM is supported by previous studies [38, 39]. LINC00152 has become a powerful prognostic biomarker in GBM patients. Although previous studies found no direct association with GBM, the remaining four lncRNAs regulate several GBM-related genes. For example, ZMIZ1-AS1 regulates several GBM-related genes, including CPEB4 and RNF43 [42, 43]. LOXL1-AS1 has been suggested to promote medulloblastoma proliferation and metastasis by activating the PI3K-AKT pathway [44]. KEGG pathways related to all five lncRNAs are shown in Supplementary Figure 1. Some studies have shown decreased RNF43 expression correlated with poor prognosis in GBM and low grade glioma (LGG) patients, while high expression of CPEB4 was associated with shorter OS rates. The differential expression of these five lncRNAs significantly correlated with CNV in GBM patients. The differential expression of another three prognostic-related lncRNAs might be caused by epigenetic changes in GBM patients, including DNA methylation, histone modification, dysregulation of transcription and/or biological defects of lncRNAs [45]. We also explored the relationship between CNV of lncRNAs and the OS rate of GBM patients using a univariate Cox regression model. All five lncRNAs may pose risk by disrupting important cancer-related biological processes that contribute to positive GBM outcomes. In order to evaluate the prognostic value of these five lncRNAs, RNA-seq data and clinical information were obtained from the CGGA database. Data derived from CGGA were set as the validation set, and we confirmed this positive correlation between expression levels of the three lncRNAs and prognosis.

The purpose of this study was to systematically identify lncRNAs that serve as prognostic biomarkers for GBM by measuring the expression of lncRNAs in GBM and their CNV. The prognostic potential of lncRNAs was evaluated, ranging from alterations at the DNA sequence level to dysregulation at the transcriptional level. We found five lncRNAs that can be used as prognostic biomarkers for GBM. Our methodology relies on the availability of multidimensional data, but. there are few large-scale multi-omics and clinical data sets for GBM. Getting more large-scale multi-omics and clinical data of GBM will further improve the predictive power of our approach.

In summary, we identified five prognostic lncRNAs associated with survival in GBM patients through a comprehensive analysis of the expression of lncRNA and their CNV. Expression of lncRNAs and/or CNA are both effective indicators for predicting survival of GBM patients and may become potential biomarkers for prognosis of GBM.

## MATERIALS AND METHODS

### RNA-Seq data

Fragments Per Kilobase of transcript per Million mapped reads (FPKM) and counts data from 167 GBM and 5 normal samples were downloaded from TCGA (https://tcga-data.nci.nih.gov/tcga/). FPKM data were converted into Transcripts Per Kilobase Million (TPM). The mRNAs with genotypes belonging to “lincRNA”, “sense_intronic”, “sense_overlapping”, “antisense”, “processed_transcript”, and “3prime_overlapping_ ncRNA” were classified as lncRNAs. Then, the FPKM expression profiles of the lncRNAs were extracted. Coding genes were determined based on their genotype classification as protein-coding, and FPKM expression profiles of coding genes were also extracted.

### TCGA data analysis

The 450k methylation profile data from 153 GBM and 2 normal tissue samples were downloaded from TCGA. Probe data of CpG-expressing NA in each sample were deleted. Cross-reactive CpG sites that were discovered by cross-reactive probes and polymorphic CpGs in the Illumina Infinium Human- Methylation 450k microarray were eliminated. Unstable genomic methylation sites were further removed. The CpGs and single nucleotide sites from sex chromosomes were eliminated. Methylation profiles of 288 GBM samples from 27k were then downloaded. Similarly, CpG probes expressing NA in each sample were removed. Methylation profiles of the CpG probes from 27k and 450k were combined and batch effects were removed using the combat function of the R package sva [46, 47].

CNV data from 418 GBM samples without species differences were downloaded from TCGA. Single nucleotide mutation data from 393 GBM samples processed using Mutect software were downloaded from TCGA [48]. Clinical follow-up data from patients associated with 599 GBM samples were downloaded from TCGA.

### Univariate survival analysis

To better classify the samples, we analyzed the effects of coding genes, CNV and methylation on the prognosis. We selected samples with a follow-up of more than 30 days and established a model using univariate Cox proportional risk regression. The threshold of *p* value was defined as 0.05.

### Multi-omics cluster analysis

Prognostic-related coding genes, CNV and methylation sites were utilized for the analysis. A total of 123 samples, covered by the three omics databases were selected. The R software package of iCluster Plus was applied to perform a multi-omics cluster analysis [49]. The iCluster Plus package can be downloaded from the Bioconductor, an open source software framework (http://www.bioconductor.org/).

### Differential analysis of lncRNA and coding genes among various subtypes

Differentially expressed lncRNAs and coding genes in various subtypes were further analyzed using the R package of DEseq2 [50]. First, genes whose average count numbers were less than one were deleted. Second, differential lncRNAs and coding genes in each subtype were screened out based on the criteria of fold-change greater than two and false discovery rate (FDR) less than 0.05 as the threshold. After ranking by the absolute values of difference multiples in each subtype, the screened lncRNAs underwent GSEA analysis [51].

### WGCNA analysis of coding genes and lncRNA with subtype differences

Using the WGCNA co-expression algorithm, we identified co-expression modules of coding genes and lncRNAs based on their differential expression profiles [51–54]. Initially, data from FPKM were converted into TPM, and the expression profiles of lncRNAs and protein-coding genes were extracted. Next, a cluster analysis was performed on these samples via hierarchical clustering. The distances between each gene and lncRNA were calculated using Pearson correlation coefficients [55] and a weighted co-expression network was constructed using the WGCNA R package [52]. The soft threshold was set at 3 to screen co-expression modules. The next step was to convert an expression matrix into an adjacency matrix, and then to convert it into a topological matrix. Based on Topological Overlap Matrix (TOM), the genes clustered using a hierarchical clustering method (average-linkage method) [56]. According to the criteria of Dynamic Tree Cut, the minimum gene number of the network module for each gene (lncRNA) was set at 30. After determining the gene module in the Dynamic Tree Cut, we calculated eigengenes of each module in turn, applied a cluster analysis to the modules and merged the nearer modules into a new one. The values of height, deepSplit and minModuleSize were set to 0.25, 2 and 30, respectively. Finally, the functionality of the modules with significant enrichment of lncRNAs was further analyzed. KEGG and GO analysis was conducted using the Cluster Profiler R package [57–60].

### CNV analysis of lncRNA

We analyzed the copy number changes of each gene from 596 cases of glioma downloaded from TCGA using GISTIC 2.0 [61, 62]. First, we extracted lncRNA copy number profiles. Copy number no less than 1 was regarded as the threshold for multicopy; no more than -1 was the threshold of copy deletion. Based on these principles, we calculated the proportion of multicopy and copy deletion of each lncRNA and investigated their distribution in the genome. Then, correlation distributions among the expression profiles of lncRNAs and copy numbers were calculated. Finally, frequent changing regions of the GBM genome were identified using the GISTIC algorithm.

### LncRNA-based prognostic biomarkers in GBM patients

To systematically identify lncRNAs with prognostic value, we analyzed copy numbers of lncRNAs with differential expression in each subtype. LncRNAs were selected only if the proportion of their CNV were more than 0.1% in each sample and differences in expression occurred among at least three subtypes. The relationships among the lncRNAs and overall survival time were analyzed using univariate survival analysis. The threshold value was set at *p* < 0.05, and 22 lncRNAs with significant prognostic value were eventually obtained. The efficacy of prognostic classification was analyzed and receiver operator characteristic (ROC) curves were drawn based on the expression of these 22 prognostic lncRNAs in each sample. LncRNAs with areas under the curve (AUC) greater than the average were screened out. Therefore, independent prognostic factors were screened using stepwise multivariate Cox regression. KEGG Pathway and GO Term enrichment analysis for each sample were conducted using gene expression profile and ssGSEA [63, 64]. The top 20 KEGG pathways and GO terms with high correlation with risk scores of the samples were further screened out.

### Validation of external data sets

To verify the prognostic roles of these CNV-related lncRNAs, RNA-seq data were downloaded from CGGA and compared with datasets from CGGA. We determined the expression profiles of lncRNAs with analyses that matched between TCGA and CCGA. We further analyzed the relationships between expression levels and prognosis. Prognosis was determined based on expression levels of the three lncRNAs.

## Supplementary Material

Supplementary Figure 1
