# Supplementary material for: Systematic identification of lncRNA-based prognostic biomarkers for glioblastoma
Source: Aging (Albany NY). 2019 Nov 6;11(21):9405–23. doi: 10.18632/aging.102393 (PMC6874448; doi:10.18632/aging.102393)
Supplement: Supplementary Figure 1 [file aging-11-102393-s001.pdf]

SUPPLEMENTARY FIGURE

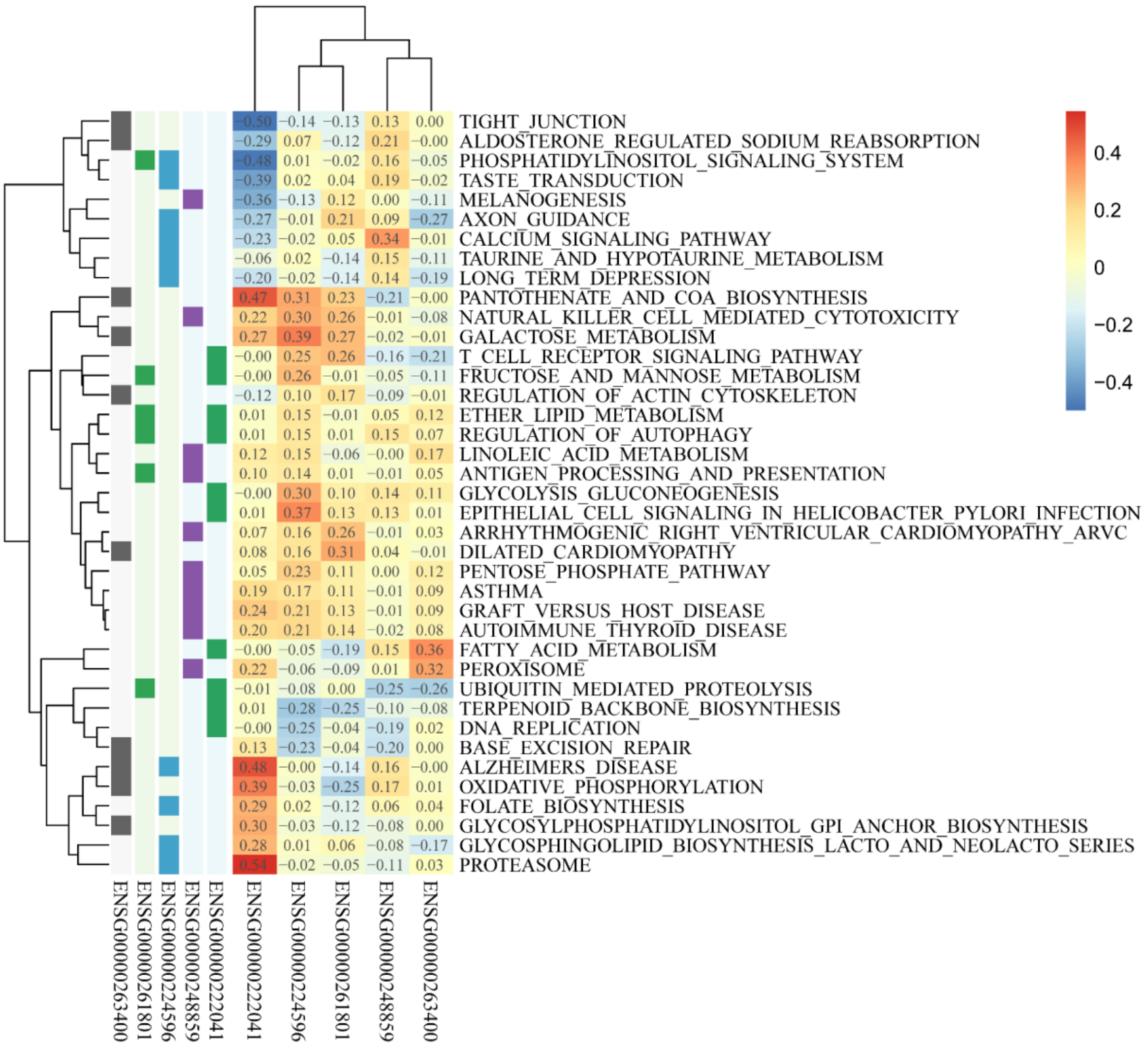

Supplementary Figure 1.

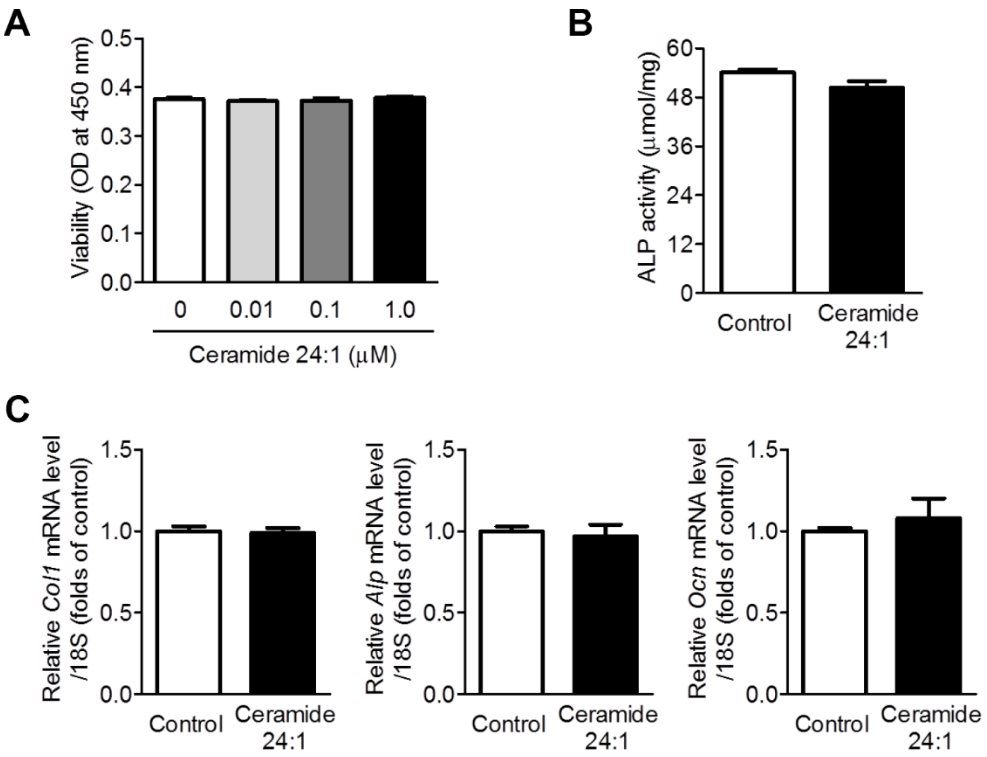

Supplementary Figure 2. No effects of ceramide 24:1 on osteoblast biology. (**A**) The viability of primary mouse calvaria osteoblasts was assessed using a Cell Counting Kit-8 assay after exposure to the indicated concentration of C24:1 for 48 hours measured. (**B**) ALP activity of calvaria osteoblasts in medium containing 50 μg/mL ascorbic acid and 10 mM β-glycerophosphate without or with 0.01 μM C24:1 for seven days. The ALP activity was normalized by total cellular protein amounts. (**C**) qRT-PCR expression analysis of osteoblast differentiation markers in calvaria osteoblasts exposed to 50 μg/mL ascorbic acid and 10 mM β-glycerophosphate without or with 0.01 μM C24:1 for seven days. Data are presented as mean ± SEM.

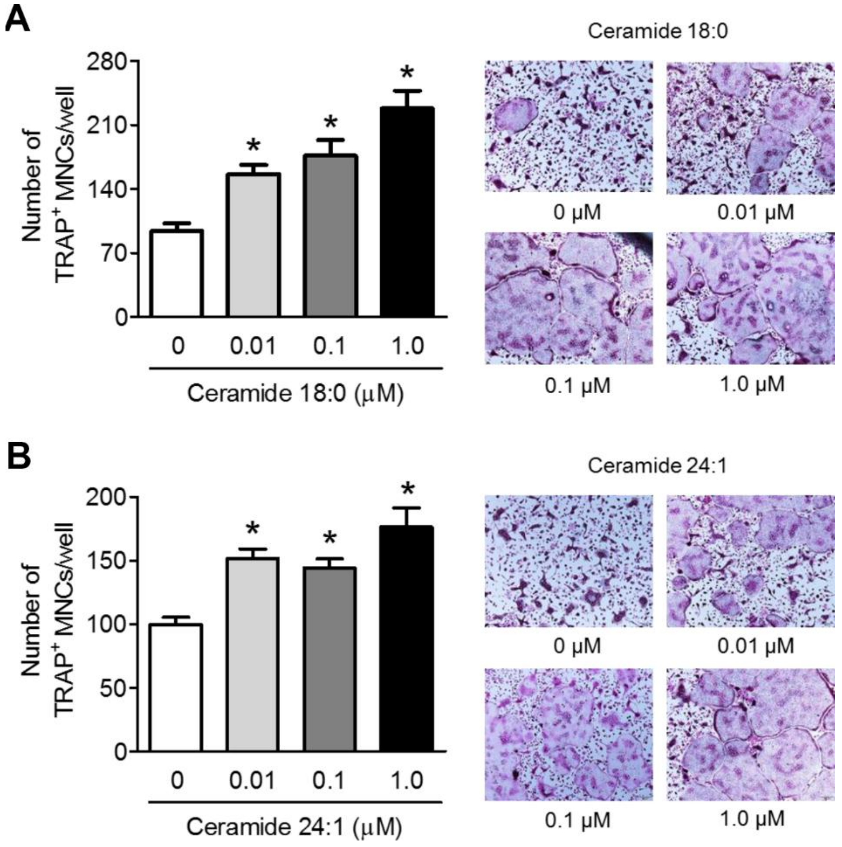

Supplementary Figure 3. Ceramide 18:0 and 24:1 stimulates osteoclast differentiation from BMMs isolated from old mice. Primary mouse BMMs were obtained from 24-month-old mice and incubated with 30 ng/mL M-CSF and 100 ng/mL RANKL in the absence or presence of the indicated concentration of C18:0 (A) and C24:1 (B) for four days. After staining cells with TRAP, the number of TRAP-positive multinucleated cells (MNCs) (≥3 nuclei/cell) was determined to assess osteoclast differentiation. *Scale bars*: 500 μm for (A) and (B). Data are presented as mean ± SEM. $^{*}P < 0.05$ vs. untreated control using the ANOVA followed by Tukey's posthoc analysis.
